# Association of chromosome 5q21.3 polymorphisms with the exploratory eye movement dysfunction in schizophrenia

**DOI:** 10.1038/srep10299

**Published:** 2015-08-05

**Authors:** Yuanlin Ma, Jun Li, Hao Yu, Lifang Wang, Tianlan Lu, Chao Pan, Yonghua Han, Dai Zhang, Weihua Yue

**Affiliations:** 1 Institute of Mental Health, The Sixth Hospital, Peking University, Beijing 100191, China; 2 Key Laboratory of Mental Health, Ministry of Health (Peking University), Beijing, 100191, China; 3 School of Life Sciences, Tsinghua University, Beijing 100084, China; 4 Peking University-Tsinghua University Joint Center for Life Sciences, Beijing 100871, China; 5 PKU-IDG/McGovern Institute for Brain Research, Peking University, Beijing, China

## Abstract

Schizophrenia patients show abnormalities in many eye movement tasks. Among them, exploratory eye movements (EEM) dysfunction seems to be specific to schizophrenia. However the mechanism of EEM disturbances in schizophrenia patients remains elusive. We investigate the relationship between EEM and single nucleotide polymorphisms (SNPs) or genes to identify susceptibility loci for EEM in schizophrenia. We firstly performed EEM test, then performed a genome-wide association study (GWAS) and gene-based association study of EEM in 128 individuals with schizophrenia and 143 healthy control subjects. Comparing to healthy controls, schizophrenia patients show significant decrease in NEF (22.99 ± 3.96 vs. 26.02 ± 5.72, P <0.001), TESL (368.78 ± 123.57 vs. 603.12 ± 178.63, P <0.001), MESL (16.86 ± 5.27 vs. 24.42 ± 6.46, P <0.001), RSS (8.22 ± 1.56 vs. 10.92 ± 1.09, P <0.001), and CSS (5.06 ± 0.97 vs. 6.64 ± 0.87, P <0.001). Five SNPs of the MAN2A1, at 5q21.3, were associated with EEM abnormalities (deceased CSS) and satisfied the criteria of GWAS significance threshold. One is localized near 5’-UTR (rs17450784) and four are in intron (rs1438663, rs17162094, rs6877440 and rs10067856) of the gene. Our findings suggest that the identified loci may control the schizophrenia-related quantitative EEM trait. And the identified gene, associated with the EEM phenotype, may lead to new insights into the etiology of schizophrenia.

Schizophrenia is a complex central nervous disease with a lifetime prevalence of 0.7% and estimated heritability of approximately 80%^1^. Lacking of pathological hallmarks, although schizophrenia is a highly prevalent mental disorder, it continues to be one of the least understood. A biological marker that is related to a genetic predisposition to schizophrenia could facilitate linkage analyses of schizophrenia and will help us to identify a major susceptibility locus for schizophrenia^2^,^3^.

Eye movement abnormalities, including smooth pursuit eye movements, saccadic eye movements and exploratory eye movements (EEM), are among the most reproducible physiological dysfunctions associated with schizophrenia^4^,^5^,^6^,^7^,^8^,^9^. Increasing evidences indicate that exploratory eye movement (EEM) may be specific to schizophrenia^10^,^11^,^12^,^13^. EEM is a method to examine the participant’s eye tracking while viewing stationary S-shaped figures. Commonly, five parameters are used to reveal the schizophrenia-related abnormalities in EEM task, including total eye scanning length (TESL), mean eye scanning length (MESL), number of eye fixations (NEF), responsive search score (RSS), and cognitive search score (CSS)^11^,^14^. Schizophrenia patients showed fewer NEF, shorter MESL, decreased RSS and CSS than controls, when performing the EEM test^11^,^14^,^15^,^16^. In most previous studies, comparing to normal individuals or patients without schizophrenic psychosis, schizophrenia patients showed significant differences in the patterns of eye tracking, the sensitivity of EEM was greater than 70% and specificity of EEM was higher than 80% for distinguishing schizophrenics from non-schizophrenics^6^,^11^,^12^,^17^. Additionally, voxel-based morphometric studies suggested that EEM abnormalities in schizophrenia could be attributed to brain structure impairments and functional disability^14^,^18^. Moreover, according to previous studies, abnormal patterns of EEM did not improve with relieved clinical symptoms of schizophrenia^15^,^19^. Based on previous study, EEM impairments(decreased NEF and RSS) were also found in the healthy siblings of schizophrenic patients^20^. Therefore, EEM dysfunction appears to be a biological marker for schizophrenia, proposed by many investigators^11^,^12^,^20^,^21^,^22^.

It is widely accepted that genetic factors play roles in pathology of schizophrenia^1^,^3^. In recent years, studies investigating the neural substrate of eye movement have pointed that abnormal smooth pursuit eye movements (SPEM) were associated with several genes, such as *COMT, ZDHHC8*, *ERBB4, RANBP1* and *NRG1*^23^,^24^,^25^,^26^,^27^,^28^. However, the underlying pathological mechanism of the EEM dysfunction in schizophrenia is largely unknown. A 10-cM resolution genome-wide linkage analysis has suggested that RSS reflects the disposition of susceptibility for schizophrenia and possible linkage between RSS and chromosome 22q11.2^29^. This study advanced the understanding of EEM dysfunction. However, the mechanism of EEM abnormalities, decreased CSS, in schizophrenia still needs to be further studied with newly developed methods.

In current study, we aims to identify chromosomal loci associated with the EEM dysfunctions in schizophrenia by combined using GWAS and gene-based association study.

## Methods

The methods were carried out in accordance with the approved guidelines.

### Ethics statement

This study was approved by the Medical Research Ethics Committee of the Institute of Mental Health, Peking University. Written informed consent was obtained from all participants.

### Subjects

We recruited 128 schizophrenia patients from the Institute of Mental Health, Peking University. All the patients satisfied diagnostic criteria for schizophrenia from the Diagnostic and Statistical Manual of Mental Disorder IV (DSM-IV), the diagnoses were made by two trained and experienced psychiatrists. Patients with a history of head injury, organic psychiatric disease, neurological disease, and drug abuse were excluded. The clinical symptoms of the patients were assessed within the same week of EEM examination by an experienced psychiatrist with Positive and Negative Syndrome Scale (PANSS). All patients received antipsychotic medications during the study, and the daily dose of antipsychotic medication was converted into chlorpromazine-equivalent dose. One hundred and forty-three healthy control subjects, without a personal history of psychiatric illness or a family history of schizophrenia spectrum disorders, were recruited from community by psychiatrists with a simple non-structured interview. The schizophrenia patients and the healthy controls were well matched for gender, age and years of education (Supplementary Table 1).

### Exploratory eye movement (EEM) test

EEM is a method to examine the participant’s eye tracking while viewing stationary S-shaped figures (Supplementary Figure 1 and Supplementary Figure 2). Measurements of total eye scanning length (TESL), mean eye scanning length (MESL), number of eye fixations (NEF), responsive search score (RSS), and cognitive search score (CSS) are followed that we descripted previously^14^.

### Genotyping

Peripheral blood samples were collected from all subjects 128 patients with schizophrenia and 143 healthy control subjects. Genomic DNA was extracted using the Qiagen QIAamp DNA Mini Kit. The genotyping of denatured samples in the first stage of the GWAS was performed on Illumina HumanHap610-Quad BeadChips, which include 620,901 SNPs and CNV probes in total, as we descripted previously^30^.

### Statistical analysis

To test whether EEM were significantly altered in schizophrenic patients, comparisons of each EEM parameter between two groups was performed using t-test. Statistical significance was set at p <0.05 (two-tailed). Statistical analysis was carried out with SPSS for windows (SPSS 17.0, SPSS Inc, Chicago, IL, USA).

In current study, using PLINK v 1.07, we performed linear regression for association analyses of EEM abnormality among schizophrenia patients. Then, to investigate the contribution of multiple SNPs to risk for EEM dysfunction in schizophrenia, each with potentially small effect, we conducted a gene-based analysis. We selected an open-source tool named Knowledge-Based Mining System for Genome-Wide Genetic Studies (KGG) (bioinfo.hku.hk/kggweb/)^31^ for the following reasons. Firstly, the KGG uses all SNPs in the gene and it combines the SNPs-related biological knowledge with the p-values of single marker analysis to produce optimal weights, which can maximize the potential power of pathway-based analysis while controlling false positive discoveries. Moreover, KGG is versatile and able to quickly produce valid pathway-based p-value without processing time-consuming permutation test and the raw genotype or phenotype. For gene-based analysis described in KGG^31^, there are two main steps: first, the SNPs were mapped to their respective genes based on their coordinates in the RefGene (hg18) data set from the UCSC database (http://genome.ucsc.edu/). And we also included the region 5 kb upstream and 5 kb downstream of each gene to account for variants in potential gene control regions. Second, for each gene, a gene-level statistics was computed using the formula in the method of improved Simes test (GATES)^31^. This step constructed weights for the gene-based test according to a priori knowledge of a particular set of SNP being found to be associated with disease. We used the default weight setting parameters in KGG, conservation score threshold 0.8, nature selection score threshold 2.0.

## Results

Table 1 shows a comparison between schizophrenia patients and healthy controls in eye movement parameters. In the retention task, there were significant differences in the three parameters of EEM (NEF, TESL and MESL) between groups, NEF (22.99 ± 3.96 *vs.* 26.02 ± 5.72), TESL (368.78 ± 123.57 *vs.* 603.12 ± 178.63), and MESL (16.86 ± 5.27 *vs.* 24.42 ± 6.46) (*P* <0.001). Moreover, during the comparison task, patients showed significant decreases in RSS (8.22 ± 1.56 *vs.* 10.92 ± 1.09) and CSS (5.06 ± 0.97 *vs.* 6.64 ± 0.87) (*P* <0.001). As shown in Supplementary Table 2, we found that parameters (TESL, MESL, NEF, RSS and CSS) of EEM were not affected by demographic characteristics (sex, age and education years), except that RSS was affected by sex. As shown in Supplementary Table 3, we found that EEM abnormalities showed no significant correlation with onset age, duration of illness, severities of illness or medications.

In this study, we performed a GWAS of 128 individuals with schizophrenia of Han Chinese using Illumina Human610-Quad BeadChips, 620,901 SNPs and copy number variation (CNV) were genotyped. No sample were excluded because of gender discordance, high genotype missing rate (>10%) or relative relationship. We excluded 101,159 SNPs with minor allele frequencies (MAF) <1%, 20656 SNPs with call rates <90%, and 438 SNPs with significant deviation from Hardy-Weinberg equilibrium (*P* < 1 × 10^−5^) in the schizophrenia patients. After stringent quality control filtering, the total genotyping rate was above 99% in the remaining individuals (128 schizophrenia patients) and 498,648 remaining autosomal SNPs.

**Figure 1:**
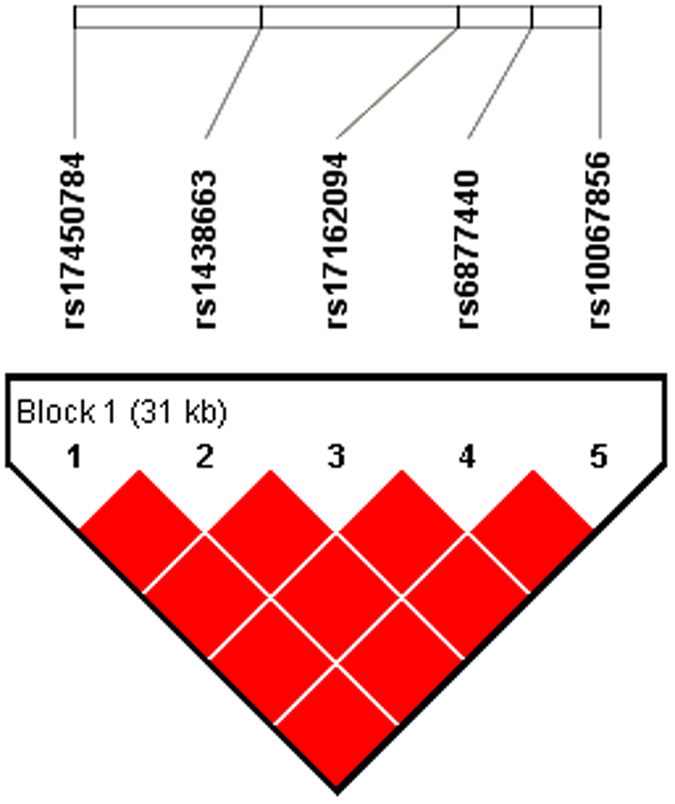
The linkage disequilibrium (LD) block structure consisted of the five SNPs located in *MAN2A1* gene. The LD block was defined by a D’ value threshold of 0.8. The color scale ranges from red to white (color intensity decreases with decreasing D’ value, and all of D’ values were  =  1). This locus was identified as one block, and the plot was generated by Haploview.

In further analysis, we tested each SNP for association with CSS using the Linear regression analysis^32^ performed with PLINK v1.07 (http://pngu.mgh.harvard.edu/~purcell/plink/) and the obtained *P* values were corrected for multiple testing by Bonferroni method. In the initial GWAS, five SNPs of gene *MAN2A1* (5q21.3) satisfied the criteria of GWAS significance threshold (Bonferroni correction *P* <0.05/498,648, or i.e., ~1.0 × 10^−7^) (Table 2 and Supplementary Figure 3). Of the five SNPs of *MAN2A1*, one was localized near 5’-UTR (rs17450784) and four were in intron (rs1438663, rs17162094, rs6877440 and rs10067856) of the gene. We consulted the dbSNP (http://www.ncbi.nlm.nih.gov/snp; accessed October 12, 2012) and HapMap (release #24, CHB; http://hapmap.ncbi.nlm.nih.gov/; accessed October 12, 2012) databases and determined the Linkage disequilibrium (LD) block using the criterion of D’ > 0.80 and Haploview version 4.0. LD was computed between every two SNPs to further analyze the haplotype structure. As shown in Fig. 1, the LD plot constructed using the five SNPs. The D’ value of each combination was = 1, therefore, these SNPs were complete linkage disequilibrium. One SNP (rs1007119) located in 2q36.1 was also significant after Bonferroni correction. In addition, twenty six SNPs gave *P* values smaller than the suggestive significant level of *P* < 1.0 × 10^−5^(df = 1) (Supplementary Table 4 and Supplementary Figure 3).

To confirm these results, we conducted a gene-based association analysis (KGG)^33^ between genes and CSS, 498,648 autosomal SNPs were mapped to their respective genes and 25349 genes were totally analyzed. Four genes, *MAN2A1*, *RPL23AP28*, *PACS2*, and *MTA1* were significantly associated with CSS, with *P* values smaller than significance threshold of Bonferroni multiple test (0.05/25349 or ~1.97 × 10-6) (Table 2 and Supplementary Table 5).

Additionally, we also tested each SNP for association with RSS, NEF,MESL and TESL using the Linear regression analysis^32^ performed with PLINK v1.07 (http://pngu.mgh.harvard.edu/~purcell/plink/), twenty seven SNPs, twenty eight SNPs ,seven SNPs and nine SNPs, respectively, gave *P* values smaller than the suggestive significant level of *P* <1.0 × 10^-5^(df = 1), while none of these SNPs satisfied the criteria of GWAS significance threshold (Bonferroni correction *P* <0.05/498,648, or i.e., ~1.0 × 10^−7^) (Supplementary Table 6, Supplementary Table 7, Supplementary Table 8 and Supplementary Table 9).

## Discussion

We found that, in the comparison task, schizophrenic patients showed decreased CSS than healthy controls, consistent with previous studies.^14^ In comparison task, two figures that differ slightly from the target figure were used to investigate how often the subject fixates on those parts of the figures that are actually different in order to confirm that they differ. CSS is obtained according to the frequency of fixation points focused on important areas of the recorded figure, and reflects abilities for fine discrimination. Series of processes involved in CSS, such as perception (visual stimulus of the S-shaped figures), memory and execution. In addition, we also found that CSS showed no significant correlation with onset age, duration of illness, severities of illness and medications (Supplementary Table 3); moreover, CSS was not affected by demographic characteristics (sex, age and education, Supplementary Table 2). Therefore, we took CSS as an intermediate phenotype and a vulnerability marker for schizophrenia in GWAS and gene-based association study.

In the initial GWAS between SNP and CSS, at 5q21.3, one validated SNP, rs17450784, was found in flanking 5’-UTR of *MAM2A1,* and another 4 validated SNPs (rs1438663, rs17162094, rs6877440 and rs10067856) were found in the intron of *MAM2A1* gene. *MAM2A1* gene encodes a glycosyl hydrolase that localizes to the Golgi and catalyzes the final hydrolytic step in the asparagine-linked oligosaccharide (N-glycan) maturation pathway. A principle function of N-glycan maturation pathway is controlling protein post-translation modification through biosynthesizing N-glycoprotein, which involves a multitude of enzymes, glycosyltransferases, and glycosidases, encoded by distinct genes. As newly synthesized glycoproteins move through the secretory pathway, the asparagine-linked glycan (N-glycan) undergoes extensive modifications involving the sequential removal and addition of sugar residues. These modifications are critical for the proper assembly, quality control and transport of glycoproteins during biosynthesis^34^. Kukuruzinska and Lennon pointed out that N-glycosylation involved in other cellular functions, including secretion, cytoskeletal organization, proliferation, and apoptosis^35^. According to previous studies, N-glycan affects the fate of neural-function related molecule; Bonnon *et al.* found that N-glycan governed the trafficking of paranodin and its selective association with contactin and neurofascin-155^36^. In the retina, accumulation of mannose rich glycosylated synaptophysin, an abundant presynaptic protein involved in synaptic vesicle recycling and neurotransmitter release, accelerated degradation of retinal synaptophysin^37^. Rosenbaum *et al.* proved that alpha-Man-II (MAN2A1) and alpha-Man-IIb function at distinct stage in maturation of Rh1, the major rhodopsin in Drosophila melanogaster photoreceptors^34^.

Akama *et al.* generated MII(*Man2a1*)/MX(*Man2a2*) double-null mice, some died between embryonic days 15.5 and 18.5, but most survived until shortly after birth and died of respiratory failure. Thus either of two is required for late embryonic and early postnatal development^38^. According previous study on Sprague-Dawley (SD) rats, although the MAN2A1 protein was either not expressed or lowly expressed in the molecular layer of the cerebral cortex and hippocampal layers, was found to be highly expressed in other areas of the brain^39^. The expression pattern and function data suggested that *MAN2A1* may play roles in the process of advanced brain function.

Interestingly, another associated gene, *PACS2*, was found through gene-based analysis (Supplementary Table 5), *PACS2* encodes phosphofurin acidic cluster sorting protein 2, which regulates the trafficking of ion channel^40^. Myhill *et al.* pointed out that PACS2 mediated calnexin subcellular distribution^41^. The alpha-glucosidases participate in glycoprotein folding mediated by calnexin and calreticulin by forming the monoglucosylated high mannose oligosaccharides required for the interaction with the chaperones^42^. Again, these results indicated that N-glycan maturation pathway may be involved in the dysfunction of EEM.

As shown in Supplementary Table 4, the *p* value of SNP rs1007119, in LOC646644 gene, satisfied the criteria of GWAS significance threshold (Bonferroni correction *p* <0.05/498,648, or i.e., ~1.0 × 10^−7^). We have checked the literatures and also made bioinformatic analysis for the potential role of this locus. We then found that the LOC646644 gene is a pseudogene of LLP homolog, long-term synaptic facilitation. However, not any more function or information could be found. The further functional exploration for this locus, especially in the EEM trait in schizophrenia, need to be explored in our future work.

We have reanalyzed the association between COMT, ERBB4 or NRG1 and EEM in schizophrenia (Supplementary Table 10, Supplementary Table 11, and Supplementary Table 12). The results showed the top five associated SNPs with CSS of *ERBB4*, *NRG1* and *COMT* respectively, while none of the SNPs of schizophrenia associated genes *COMT*, *ERBB4* or *NRG1* was satisfied the criteria of GWAS significance threshold (Bonferroni correction *P* < 0.05/498,648, or i.e., ~1.0 × 10^−7^). This may result from following reasons, firstly, the association studies on relationship between gene and abnormal eye movements focused on SPEM, while in our study we took EEM as a biological maker for linkage study. Secondly, most of the previous studies were performed in Japanese population or Korean population. Last but not least, the sample of this study might not be large enough to identify positive association of *COMT*, *ERBB4* or *NRG1* with EEM impairments.

In summery we performed GWAS and gene-based association study for exploratory eye movement in schizophrenia of Han Chinese population, and identified a new susceptibility locus at 5q21.3. Although further replication studies in larger samples are needed, findings from this study may be useful in guiding future studies into the etiology of schizophrenia.

## Additional Information

**How to cite this article**: Ma, Y. *et al.* Association of chromosome 5q21.3 polymorphisms with the exploratory eye movement dysfunction in schizophrenia. *Sci. Rep.* **5**, 10299; doi: 10.1038/srep10299 (2015).

## Supplementary Material

Supplementary Information

## Figures and Tables

**Table 1:** 

Parameters	Patients (n=128)	Controls (n=143)	*t*-value	*P*-value
RSS	8.22 ± 1.56^a^	10.92 ± 1.09	16.36	<0.001^b^
CSS	5.06 ± 0.97	6.64 ± 0.87	14.17	<0.001
NEF	22.99 ± 3.96	26.02 ± 5.72	5.11	<0.001
TESL	368.78 ± 123.57	603.12 ± 178.63	12.66	<0.001
MESL	16.86 ± 5.27	24.42 ± 6.46	10.47	<0.001

Comparison of EEM parameters (NEF, TESL, MESL, RSS, and CSS) between schizophrenia patients and healthy controls. RSS, responsive search score; CSS, cognitive search score; NEF, number of eye fixations; TESL, total eye scanning length; MESL, mean eye scanning length.

^a^Mean ± standard deviation.

^b^Two-sample t-test.

**Table 2:** 

Chr	SNP	Position^a^	Location	Beta	SE	R^2^	T	*P*	Gene-symbol	Gene-*P*value	Gene-Significant^b^
5	rs17450784	109044525	flanking_5’UTR	−3.63	0.6326	0.2059	−5.738	6.64 × 10^−8^	MAN2A1	1.07 × 10^−11^	Yes
5	rs1438663	109055750	intron	−3.63	0.6326	0.2059	−5.738	6.64 × 10^−8^	MAN2A1	1.07 × 10^−11^	Yes
5	rs17162094	109067672	intron	−3.63	0.6326	0.2059	−5.738	6.64 × 10^−8^	MAN2A1	1.07 × 10^−11^	Yes
5	rs6877440	109071966	intron	−3.63	0.6326	0.2059	−5.738	6.64 × 10^−8^	MAN2A1	1.07 × 10^−11^	Yes
5	rs10067856	109076032	intron	−3.63	0.6326	0.2059	−5.738	6.64 × 10^−8^	MAN2A1	1.07 × 10^−11^	Yes
5	rs12519496	109242946	flanking_3’UTR	−3.068	0.5568	0.1929	−5.51	1.92 × 10^−7^	MAN2A1	1.07 × 10^−11^	Yes
5	rs245243	109258634	flanking_3’UTR	−2.706	0.5355	0.1674	−5.053	1.48 × 10^−6^	MAN2A1	1.07 × 10^−11^	Yes

Association results between SNP/Gene and CSS. Chr., chromosome; SNP, single neucleotide polymorphism; Beta, regression coefficient; SE, standard error; R^2^, regression r-squared; T, Wald test (based on t-distribtion); *P*, Wald test asymptotic p-value.

^a^Genomic position (in the UCSC March 2006 human reference sequence, hg18).

^b^Significance threshold of 0.05/25349 or ~1.97 × 10^−6^.
