# Supplementary material for: Association of chromosome 5q21.3 polymorphisms with the exploratory eye movement dysfunction in schizophrenia
Source: Sci Rep. 2015 Aug 5;5:10299. doi: 10.1038/srep10299 (PMC4533163; doi:10.1038/srep10299)
Supplement: Supplementary Information [file srep10299-s1.doc]

**Association of chromosome 5q21.3 polymorphisms with the exploratory eye movement dysfunction in schizophrenia**

Yuanlin Ma, Jun Li, Hao Yu, Lifang Wang, Tianlan Lu, Chao Pan, Yonghua Han, Weihua Yue & Dai Zhang

**SUPPLEMENTARY MATERIALS INDEX**

**Supplementary Figure 1. Figures in the exploratory eye movement task.**

**Supplementary Figure 2. Figures for responsive score calculation.**

**Supplementary Figure 3. Manhattan plot for GWAS findings in 128 individuals with schizophrenia.**

**Supplementary Table 1. Demographic and clinical data of schizophrenia patients and healthy controls.**

**Supplementary Table 2. Effects of sex, age and education on EEM parameters.**

**Supplementary Table 3. Effects of clinical profiles on EEM impairments.**

**Supplementary Table 4.** **The remaining 19 SNPs of association results with CSS.**

**Supplementary Table 5.** **The remaining 3 genes of association results with CSS.**

**Supplementary Table 6.** **Association results between SNP and RSS.**

**Supplementary Table 7.** **Association results between SNP and NEF.**

**Supplementary Table 8.** **Association results between SNP and MESL.**

**Supplementary Table 9.** **Association results between SNP and TESL.**

**Supplementary Table 10. Top Five SNPs of *ERBB4* associated with CSS.**

**Supplementary Table 11. Top Five SNPs of *NRG1* associated with CSS.**

**Supplementary Table 12. Top Five SNPs of *COMT* associated with CSS.**


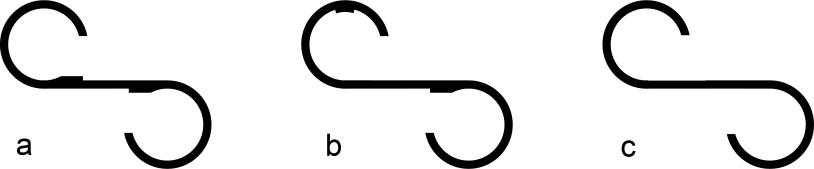


**Supplementary Figure 1. Figures in the exploratory eye movement task.**

(a) The original target figure. (b, c) Two figures that slightly differ from the target.


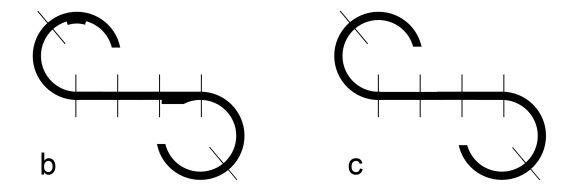


**Supplementary Figure 2. Figures for responsive score calculation.**

Both figure S1. b and c are divided into seven sections.


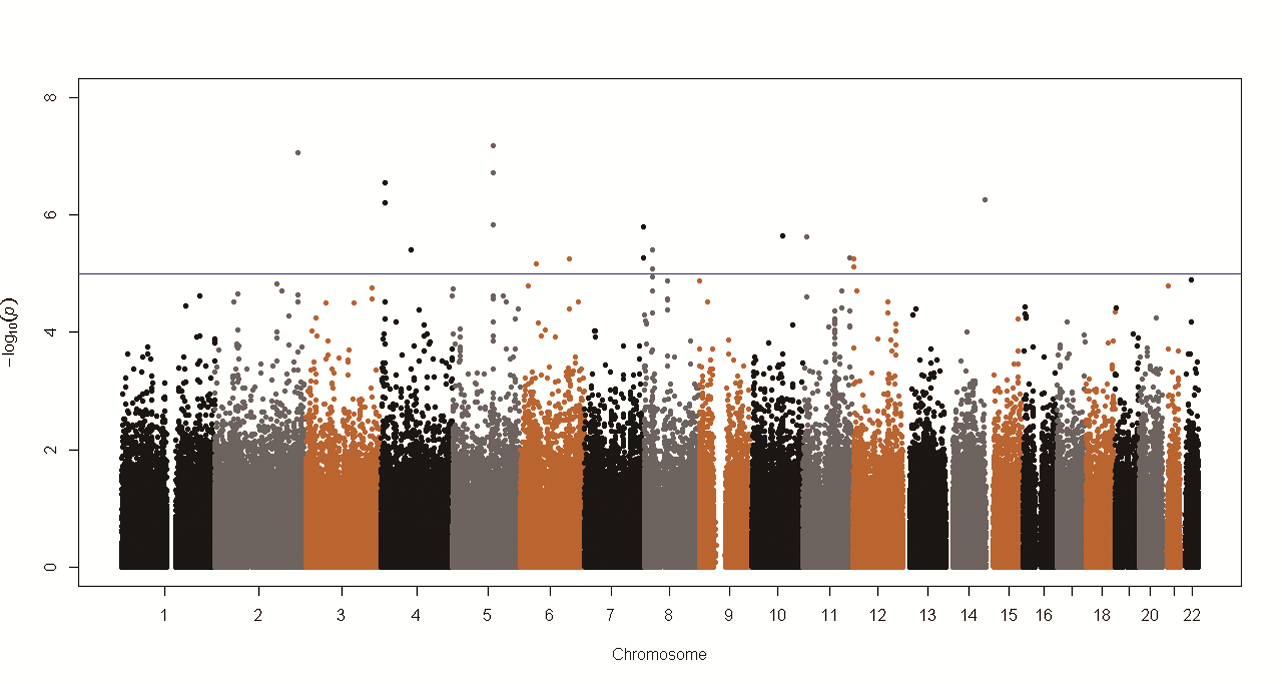


**Supplementary Figure 3. Manhattan plot for GWAS findings in 128 individuals with schizophrenia.**

Manhattan plot of GWAS results from 498,648 SNPs tested for association with cognitive search score (CSS) in 128 individuals with schizophrenia. On the y-axis is −log10 (*p*). The blue line denotes the *p*-value of 1.0×10^-5^.

**Supplementary Table 1. Demographic and clinical data of schizophrenia patients and healthy controls**

| Participant characteristics | Patients  (n=128) | Controls  (n=143) | *p* value |
| --- | --- | --- | --- |
| Male/female | 66/62 | 76/67 | 0.794^a^ |
| Age (years) | 29.17±8.81^c^ | 28.04±5.83 | 0.220^b^ |
| Education (years) | 13.09±2.78 | 12.74±2.43 | 0.270^b^ |
| Age at onset (years) | 22.75±6.78 |  |  |
| Duration of illness (months) | 77.09±78.41 |  |  |
| Drug (mg/day)^d^ | 503.09±277.82 |  |  |
| PANSS^e^ positive score | 22.70±7.08 |  |  |
| PANSS negative score | 19.40±6.92 |  |  |
| PANSS total score | 76.61±17.62 |  |  |

^a^Pearson Chi-square test

^b^Two sample t-test

^c^Mean±standard deviation

^d^Chlorpromazine-equivalent dose

^e^Positive and Negative Syndrome Scale

**Supplementary Table 2. Effects of sex, age and education on EEM parameters.**

| dependent variable | Sex | | |  | Age level | | |  | Education level | | |
| --- | --- | --- | --- | --- | --- | --- | --- | --- | --- | --- | --- |
|  | df | F | *p*^a^ |  | df | F | *p*^a^ |  | df | F | *p*^a^ |
| TESL | 1 | 1.659 | 0.199 |  | 3 | 0.236 | 0.871 |  | 2 | 0.260 | 0.771 |
| MESL | 1 | 3.612 | 0.058 |  | 3 | 0.503 | 0.681 |  | 2 | 0.097 | 0.907 |
| NEF | 1 | 0.003 | 0.959 |  | 3 | 1.599 | 0.190 |  | 2 | 0.050 | 0.951 |
| CSS | 1 | 0.856 | 0.356 |  | 3 | 1.387 | 0.247 |  | 2 | 0.340 | 0.712 |
| RSS | 1 | 9.591 | **0.002** |  | 3 | 1.902 | 0.130 |  | 2 | 1.080 | 0.341 |

TESL total eye scanning length; MESL mean eye scanning length; NEF number of eye fixations; RSS responsive search score; CSS cognitive search score.

^a^ Two-way ANOVA

**Supplementary Table 3. Effects of clinical profiles on EEM impairments.**

| dependent variable | df | F | *p*^a^ |
| --- | --- | --- | --- |
| TESL | 4 | 0.342 | 0.849 |
| MESL | 4 | 0.025 | 0.999 |
| NEF | 4 | 2.183 | 0.075 |
| RSS | 4 | 2.043 | 0.092 |
| CSS | 4 | 0.258 | 0.904 |

TESL total eye scanning length; MESL mean eye scanning length; NEF number of eye fixations; RSS responsive search score; CSS cognitive search score.

^a^ Multi-regression analysis in onset age, duration of illness, severities of illness and medications.

| **Supplementary Table 4.** **The remaining 19 SNPs of association results with CSS** | | | | | | | | | |
| --- | --- | --- | --- | --- | --- | --- | --- | --- | --- |
| Chr. | SNP | Gene-symbol | Position^a^ | Location | Beta | SE | R^2^ | T | *P* |
| 2 | rs1007119 | LOC646644 | 222630768 | flanking_3’UTR | -1.981 | 0.3488 | 0.2026 | -5.68 | **8.71×10^-8^** |
| 4 | rs4916646 | HS3ST1 | 11078828 | flanking_5’UTR | -2.125 | 0.3917 | 0.1882 | -5.425 | 2.82×10^-7^ |
| 4 | rs1495155 | HS3ST1 | 11103384 | flanking_5’UTR | -2.219 | 0.4227 | 0.1783 | -5.25 | 6.21×10^-7^ |
| 4 | rs2867695 | PRDM8 | 81292244 | flanking_5’UTR | -1.412 | 0.2925 | 0.155 | -4.827 | 3.90×10^-6^ |
| 4 | rs3755913 | PRDM8 | 81324786 | intron | -1.412 | 0.2925 | 0.155 | -4.827 | 3.90×10^-6^ |
| 5 | rs956882 | LOC728342 | 116903262 | intron | -2.952 | 0.5519 | 0.3834 | -5.348 | 2.71×10^-6^ |
| 6 | rs12197554 | TMEM200A | 130807433 | flanking_3’UTR | -3.112 | 0.6563 | 0.1514 | -4.742 | 5.63×10^-6^ |
| 6 | rs12214723 | TAF8 | 42101666 | flanking_5’UTR | -3.726 | 0.7925 | 0.1492 | -4.701 | 6.68×10^-6^ |
| 7 | rs7797990 | tcag7.1213 | 155697840 | intron | -3.548 | 0.7039 | 0.1678 | -5.04 | 1.58×10^-6^ |
| 7 | rs4716593 | tcag7.1213 | 155704176 | intron | -3.106 | 0.6542 | 0.1507 | -4.747 | 5.47×10^-6^ |
| 8 | rs11778693 | KIAA1967 | 22518797 | 5’UTR | -1.249 | 0.259 | 0.1548 | -4.823 | 3.98×10^-6^ |
| 8 | rs11136092 | KIAA1967 | 22522433 | intron | -1.249 | 0.259 | 0.1548 | -4.823 | 3.98×10^-6^ |
| 8 | rs4242434 | BIN3 | 22557775 | flanking_3’UTR | -1.216 | 0.2617 | 0.1453 | -4.647 | 8.32×10^-6^ |
| 10 | rs10882097 | LOC642666 | 82558664 | flanking_3’UTR | -1.228 | 0.248 | 0.1618 | -4.952 | 2.29×10^-6^ |
| 11 | rs9919625 | GALNTL4 | 11549487 | intron | -1.397 | 0.2823 | 0.1617 | -4.949 | 2.32×10^-6^ |
| 11 | rs663734 | LOC399965 | 124490065 | flanking_3’UTR | -2.254 | 0.4742 | 0.1511 | -4.754 | 5.32×10^-6^ |
| 12 | rs3794288 | CACNA1C | 2659482 | intron | -1.566 | 0.33 | 0.1506 | -4.745 | 5.51×10^-6^ |
| 12 | rs2238096 | CACNA1C | 2620174 | intron | -1.42 | 0.3044 | 0.1463 | -4.666 | 7.69×10^-6^ |
| 14 | rs6576086 | LOC647310 | 104944189 | intron | -4.738 | 0.8984 | 0.1797 | -5.274 | 5.57×10^-7^ |

Chr., chromosome; SNP, single neucleotide polymorphism; Beta, regression coefficient; SE, standard error; R^2^, regression r-squared; T, Wald test (based on t-distribtion ); *P*, Wald test asymptotic p-value.

^a^Genomic position (in the UCSC March 2006 human reference sequence, hg18)

| **Supplementary Table 5.** **The remaining 3 genes of association results with CSS.** | | | |
| --- | --- | --- | --- |
| Chr. | Gene-symbol | Gene-*P* value | Gene-significant^a^ |
| 2 | RPL23AP28 | 5.01×10^-7^ | Yes |
| 14 | MTA1 | 1.71×10^-6^ | Yes |
| 14 | PACS2 | 5.57×10^-7^ | Yes |

Chr., Chromosome.

^a^Significance threshold of 0.05/25349 or ~1.97×10^-6^

**Supplementary Table 6.** **Association results between SNP and RSS.**

| Chr. | SNP | Position^a^ | Beta | SE | R^2^ | T | *P* |
| --- | --- | --- | --- | --- | --- | --- | --- |
| 2 | rs1007119 | 222630768 | -2.907 | 0.5717 | 0.1692 | -5.085 | 1.28×10^-6^ |
| 3 | rs7635966 | 129702914 | -5.968 | 1.283 | 0.1465 | -4.651 | 8.23×10^-6^ |
| 4 | rs6554340 | 56822605 | -1.476 | 0.2983 | 0.1616 | -4.947 | 2.34×10^-6^ |
| 5 | rs17450784 | 109044525 | -5.089 | 1.047 | 0.1569 | -4.862 | 3.36×10^-6^ |
| 5 | rs1438663 | 109055750 | -5.089 | 1.047 | 0.1569 | -4.862 | 3.36×10^-6^ |
| 5 | rs17162094 | 109067672 | -5.089 | 1.047 | 0.1569 | -4.862 | 3.36×10^-6^ |
| 5 | rs6877440 | 109071966 | -5.089 | 1.047 | 0.1569 | -4.862 | 3.36×10^-6^ |
| 5 | rs10067856 | 109076032 | -5.089 | 1.047 | 0.1569 | -4.862 | 3.36×10^-6^ |
| 6 | rs1889399 | 20213256 | -3.488 | 0.6688 | 0.1764 | -5.216 | 7.23×10^-7^ |
| 6 | rs12214723 | 42101666 | -6.202 | 1.274 | 0.1582 | -4.866 | 3.33×10^-6^ |
| 6 | rs12197554 | 130807433 | -4.929 | 1.056 | 0.1475 | -4.669 | 7.62×10^-6^ |
| 8 | rs11778693 | 22518797 | -1.949 | 0.418 | 0.1462 | -4.663 | 7.76×10^-6^ |
| 8 | rs11136092 | 22522433 | -1.949 | 0.418 | 0.1462 | -4.663 | 7.76×10^-6^ |
| 8 | rs4077341 | 23018293 | -1.693 | 0.3641 | 0.1454 | -4.649 | 8.23×10^-6^ |
| 8 | rs10109541 | 513920 | -2.288 | 0.494 | 0.1444 | -4.631 | 8.89×10^-6^ |
| 8 | rs2977795 | 6697215 | -1.734 | 0.3761 | 0.1444 | -4.611 | 9.70×10^-6^ |
| 9 | rs279910 | 965112 | -1.571 | 0.323 | 0.1571 | -4.865 | 3.33×10^-6^ |
| 9 | rs861189 | 22175252 | -5.954 | 1.28 | 0.1455 | -4.651 | 8.18×10^-6^ |
| 11 | rs9919625 | 11549487 | -2.269 | 0.4523 | 0.1654 | -5.016 | 1.73×10^-6^ |
| 12 | rs10507017 | 91689067 | -6.212 | 1.271 | 0.1584 | -4.889 | 3.00×10^-6^ |
| 12 | rs11047510 | 8881183 | -5.089 | 1.047 | 0.1569 | -4.862 | 3.36×10^-6^ |
| 13 | rs9599293 | 34063390 | -3.033 | 0.6092 | 0.1633 | -4.978 | 2.05×10^-6^ |
| 14 | rs6576086 | 104944189 | -7.698 | 1.439 | 0.1839 | -5.35 | 3.96×10^-7^ |
| 16 | rs4783860 | 53562822 | -3.196 | 0.6802 | 0.1481 | -4.699 | 6.70×10^-6^ |
| 18 | rs11662586 | 75817159 | -3.632 | 0.7829 | 0.1449 | -4.64 | 8.56×10^-6^ |
| 19 | rs1661178 | 49738192 | -2.528 | 0.5178 | 0.158 | -4.882 | 3.09×10^-6^ |
| 19 | rs2043599 | 61142861 | -3.748 | 0.7926 | 0.1497 | -4.729 | 5.91×10^-6^ |

Chr., chromosome; SNP, single neucleotide polymorphism; Beta, regression coefficient; SE, standard error; R^2^, regression r-squared; T, Wald test (based on t-distribtion ); *P*, Wald test asymptotic p-value.

^a^Genomic position (in the UCSC March 2006 human reference sequence, hg18)

**Supplementary Table 7.** **Association results between SNP and NEF.**

| Chr. | SNP | Position^a^ | Beta | SE | R^2^ | T | *P* |
| --- | --- | --- | --- | --- | --- | --- | --- |
| 1 | rs12410615 | 57004579 | -4.505 | 0.9049 | 0.1633 | -4.979 | 2.04×10^-6^ |
| 2 | rs12622528 | 107015872 | -7.531 | 1.522 | 0.1616 | -4.948 | 2.33×10^-6^ |
| 2 | rs13396519 | 10957597 | -8.753 | 1.812 | 0.1552 | -4.83 | 3.85×10^-6^ |
| 2 | rs7598737 | 81038280 | -4.596 | 0.9791 | 0.1478 | -4.694 | 6.85×10^-6^ |
| 2 | rs10203140 | 52114256 | -16.64 | 3.552 | 0.1474 | -4.686 | 7.08×10^-6^ |
| 2 | rs1032833 | 179565022 | -10.76 | 2.304 | 0.1467 | -4.672 | 7.48×10^-6^ |
| 3 | rs7635966 | 129702914 | -16.91 | 3.557 | 0.1521 | -4.753 | 5.37×10^-6^ |
| 4 | rs1870653 | 115617649 | -12.67 | 2.72 | 0.146 | -4.659 | 7.91×10^-6^ |
| 5 | rs17450784 | 109044525 | -15.17 | 2.866 | 0.1807 | -5.292 | 5.15×10^-7^ |
| 5 | rs1438663 | 109055750 | -15.17 | 2.866 | 0.1807 | -5.292 | 5.15×10^-7^ |
| 5 | rs17162094 | 109067672 | -15.17 | 2.866 | 0.1807 | -5.292 | 5.15×10^-7^ |
| 5 | rs6877440 | 109071966 | -15.17 | 2.866 | 0.1807 | -5.292 | 5.15×10^-7^ |
| 5 | rs10067856 | 109076032 | -15.17 | 2.866 | 0.1807 | -5.292 | 5.15×10^-7^ |
| 5 | rs17108911 | 148263515 | -8.022 | 1.699 | 0.1493 | -4.721 | 6.11×10^-6^ |
| 5 | rs1661068 | 3266396 | -10.04 | 2.176 | 0.1436 | -4.614 | 9.51×10^-6^ |
| 6 | rs12214723 | 42101666 | -17.14 | 3.546 | 0.1565 | -4.834 | 3.81×10^-6^ |
| 6 | rs12197554 | 130807433 | -13.78 | 2.933 | 0.149 | -4.696 | 6.82×10^-6^ |
| 6 | rs12198650 | 153761678 | -16.64 | 3.552 | 0.1474 | -4.686 | 7.08×10^-6^ |
| 6 | rs961846 | 153779588 | -16.64 | 3.552 | 0.1474 | -4.686 | 7.08×10^-6^ |
| 6 | rs9479600 | 153788297 | -16.64 | 3.552 | 0.1474 | -4.686 | 7.08×10^-6^ |
| 9 | rs861189 | 22175252 | -17.16 | 3.533 | 0.1567 | -4.857 | 3.43×10^-6^ |
| 9 | rs279910 | 965112 | -4.17 | 0.9045 | 0.1434 | -4.611 | 9.65×10^-6^ |
| 10 | rs4608000 | 109500085 | -8.081 | 1.663 | 0.1567 | -4.859 | 3.42×10^-6^ |
| 10 | rs1710278 | 127728908 | -7.339 | 1.583 | 0.1448 | -4.636 | 8.68×10^-6^ |
| 12 | rs10507017 | 91689067 | -17.42 | 3.523 | 0.1614 | -4.944 | 2.37×10^-6^ |
| 14 | rs6576086 | 104944189 | -21.63 | 3.987 | 0.1882 | -5.426 | 2.81×10^-7^ |
| 18 | rs11662586 | 75817159 | -10.04 | 2.176 | 0.1436 | -4.614 | 9.51×10^-6^ |
| 19 | rs2043599 | 61142861 | -10.48 | 2.199 | 0.1516 | -4.764 | 5.11×10^-6^ |

Chr., chromosome; SNP, single neucleotide polymorphism; Beta, regression coefficient; SE, standard error; R^2^, regression r-squared; T, Wald test (based on t-distribtion ); *P*, Wald test asymptotic p-value.

^a^Genomic position (in the UCSC March 2006 human reference sequence, hg18)

**Supplementary Table 8.** **Association results between SNP and MESL.**

| Chr. | SNP | Position^a^ | Beta | SE | R^2^ | T | *P* |
| --- | --- | --- | --- | --- | --- | --- | --- |
| 6 | rs9399510 | 145227335 | -7.765 | 1.581 | 0.1596 | -4.911 | 2.73×10^-6^ |
| 6 | rs9403607 | 145139812 | -6.809 | 1.43 | 0.1514 | -4.761 | 5.17×10^-6^ |
| 6 | rs9390237 | 145215071 | -6.246 | 1.321 | 0.1497 | -4.729 | 5.92×10^-6^ |
| 12 | rs2111902 | 107802876 | 4.428 | 0.8114 | 0.1899 | 5.457 | 2.44×10^-7^ |
| 12 | rs3825251 | 107812113 | 4.165 | 0.826 | 0.1668 | 5.043 | 1.55×10^-6^ |
| 12 | rs6539460 | 107808607 | 3.973 | 0.8237 | 0.1548 | 4.824 | 3.96×10^-6^ |
| 12 | rs3741775 | 107807732 | 4.068 | 0.8501 | 0.1527 | 4.785 | 4.67×10^-6^ |

Chr., chromosome; SNP, single neucleotide polymorphism; Beta, regression coefficient; SE, standard error; R^2^, regression r-squared; T, Wald test (based on t-distribtion ); *P*, Wald test asymptotic p-value.

^a^Genomic position (in the UCSC March 2006 human reference sequence, hg18)

**Supplementary Table 9.** **Association results between SNP and TESL.**

| Chr. | SNP | Position^a^ | Beta | SE | R^2^ | T | *P* |
| --- | --- | --- | --- | --- | --- | --- | --- |

| 1 | rs667153 | 30698539 | -80.91 | 19.57 | 0.1186 | -4.134 | 4.30×10^-6^ |
| --- | --- | --- | --- | --- | --- | --- | --- |
| 2 | rs1860762 | 229648879 | -78.5 | 19.48 | 0.1134 | -4.039 | 5.40×10^-6^ |
| 3 | rs9809064 | 54191157 | -81.56 | 20.03 | 0.1155 | -4.072 | 1.50×10^-6^ |
| 5 | rs1533106 | 126115611 | -83.03 | 18.28 | 0.1397 | -4.542 | 2.80×10^-6^ |
| 10 | rs7899719 | 77118441 | 83.24 | 20.43 | 0.1155 | 4.073 | 1.10×10^-6^ |
| 11 | rs7928931 | 82938269 | -87.52 | 19.75 | 0.1339 | -4.431 | 1.00×10^-5^ |
| 12 | rs2122661 | 96548786 | 97.35 | 21.46 | 0.1394 | 4.536 | 3.10×10^-6^ |
| 18 | rs4797559 | 11573447 | 79.27 | 19.21 | 0.1182 | 4.126 | 6.20×10^-6^ |
| 20 | rs721424 | 20287310 | -90.94 | 20.33 | 0.1361 | -4.473 | 6.90×10^-6^ |

Chr., chromosome; SNP, single neucleotide polymorphism; Beta, regression coefficient; SE, standard error; R^2^, regression r-squared; T, Wald test (based on t-distribtion ); *P*, Wald test asymptotic p-value.

^a^Genomic position (in the UCSC March 2006 human reference sequence, hg18)

**Supplementary Table 10. Top Five SNPs of *ERBB4* associated with CSS.**

| Chr. | SNP | Position^a^ | Beta | SE | R^2^ | T | *P* |
| --- | --- | --- | --- | --- | --- | --- | --- |
| 2 | rs7594456 | 212111679 | -0.7792 | 0.2671 | 0.0628 | -2.917 | 0.004177 |
| 2 | rs10932380 | 212098595 | -0.764 | 0.2664 | 0.06081 | -2.867 | 0.004846 |
| 2 | rs13030304 | 211991391 | -1.427 | 0.4988 | 0.06056 | -2.861 | 0.004935 |
| 2 | rs16848520 | 212990094 | -4.664 | 1.654 | 0.05895 | -2.821 | 0.005563 |
| 2 | rs13387495 | 212993302 | -4.664 | 1.654 | 0.05895 | -2.821 | 0.005563 |

Chr., chromosome; SNP, single neucleotide polymorphism; Beta, regression coefficient; SE, standard error; R^2^, regression r-squared; T, Wald test (based on t-distribtion ); *P*, Wald test asymptotic p-value.

^a^Genomic position (in the UCSC March 2006 human reference sequence, hg18)

**Supplementary Table 11. Top Five SNPs of *NRG1* associated with CSS.**

| Chr. | SNP | | Position^a^ | | | Beta | | SE | R^2^ | T | | *P* | |
| --- | --- | --- | --- | --- | --- | --- | --- | --- | --- | --- | --- | --- | --- |
| 8 | | rs10092449 | | 32682905 | -2.161 | | 1.195 | | 0.02512 | | -1.809 | | 0.0728 |
| 8 | | rs12546380 | | 32655462 | 0.3258 | | 0.2054 | | 0.01943 | | 1.586 | | 0.1151 |
| 8 | | rs4602844 | | 32571440 | -0.6983 | | 0.4927 | | 0.01557 | | -1.417 | | 0.1589 |
| 8 | | rs3757934 | | 32728334 | 2.391 | | 1.691 | | 0.01549 | | 1.413 | | 0.16 |
| 8 | | rs2439300 | | 32552646 | 0.4009 | | 0.3018 | | 0.0137 | | 1.328 | | 0.1865 |

Chr., chromosome; SNP, single neucleotide polymorphism; Beta, regression coefficient; SE, standard error; R^2^, regression r-squared; T, Wald test (based on t-distribtion ); *P*, Wald test asymptotic p-value.

^a^Genomic position (in the UCSC March 2006 human reference sequence, hg18)

**Supplementary Table 12. Top Five SNPs of *COMT* associated with CSS.**

| Chr. | SNP | | | Position^a^ | | Beta | | SE | | R^2^ | | T | *P* | |
| --- | --- | --- | --- | --- | --- | --- | --- | --- | --- | --- | --- | --- | --- | --- |
| 22 | | rs737866 | 18310109 | | -0.5106 | | 0.2313 | | 0.03694 | | -2.207 | | | 0.0291 |
| 22 | | rs4646316 | 18332132 | | -0.5025 | | 0.2288 | | 0.03658 | | -2.196 | | | 0.02992 |
| 22 | | 500437 | 18330763 | | -0.4133 | | 0.2283 | | 0.02535 | | -1.81 | | | 0.07263 |
| 22 | | rs4646312 | 18328337 | | -0.4021 | | 0.2293 | | 0.02365 | | -1.754 | | | 0.08184 |
| 22 | | rs2239393 | 18330428 | | -0.4021 | | 0.2293 | | 0.02365 | | -1.754 | | | 0.08184 |

Chr., chromosome; SNP, single neucleotide polymorphism; Beta, regression coefficient; SE, standard error; R^2^, regression r-squared; T, Wald test (based on t-distribtion ); *P*, Wald test asymptotic p-value.

^a^Genomic position (in the UCSC March 2006 human reference sequence, hg18)
